# Mutation of a conserved anthranilate phosphoribosyltransferase active site residue supports tryptophan biosynthesis *in vivo*

**DOI:** 10.17912/micropub.biology.001925

**Published:** 2025-12-15

**Authors:** Stephen Klepin, Brooke Michalik, Lorraine Pillus, Jennifer K. Chik

**Affiliations:** 1 Department of Molecular Biology, University of California San Diego, La Jolla, California, United States; 2 Department of Chemistry and Biochemistry, California Polytechnic State University, San Luis Obispo, California, United States

## Abstract

Enzymes contributing to amino acid metabolism are among the most ancient in the proteome. A growing appreciation that many of these proteins have evolved to contribute multiple, distinct functions has led to an increased focus on defining such moonlighting molecules and their diverse roles. A focus on the metabolic enzyme anthranilate phosphoribosyltransferase, encoded by *TRP4* in *Saccharomyces cerevisiae* , revealed that Ser121, identified in structural studies as critical for substrate binding, is not required for two documented *in vivo* functions. These findings add functional complexity to the Trp4 active site beyond crystallographic characterization.

**Figure 1. Mutation of Trp4-S121 supports Trp4 activity:**
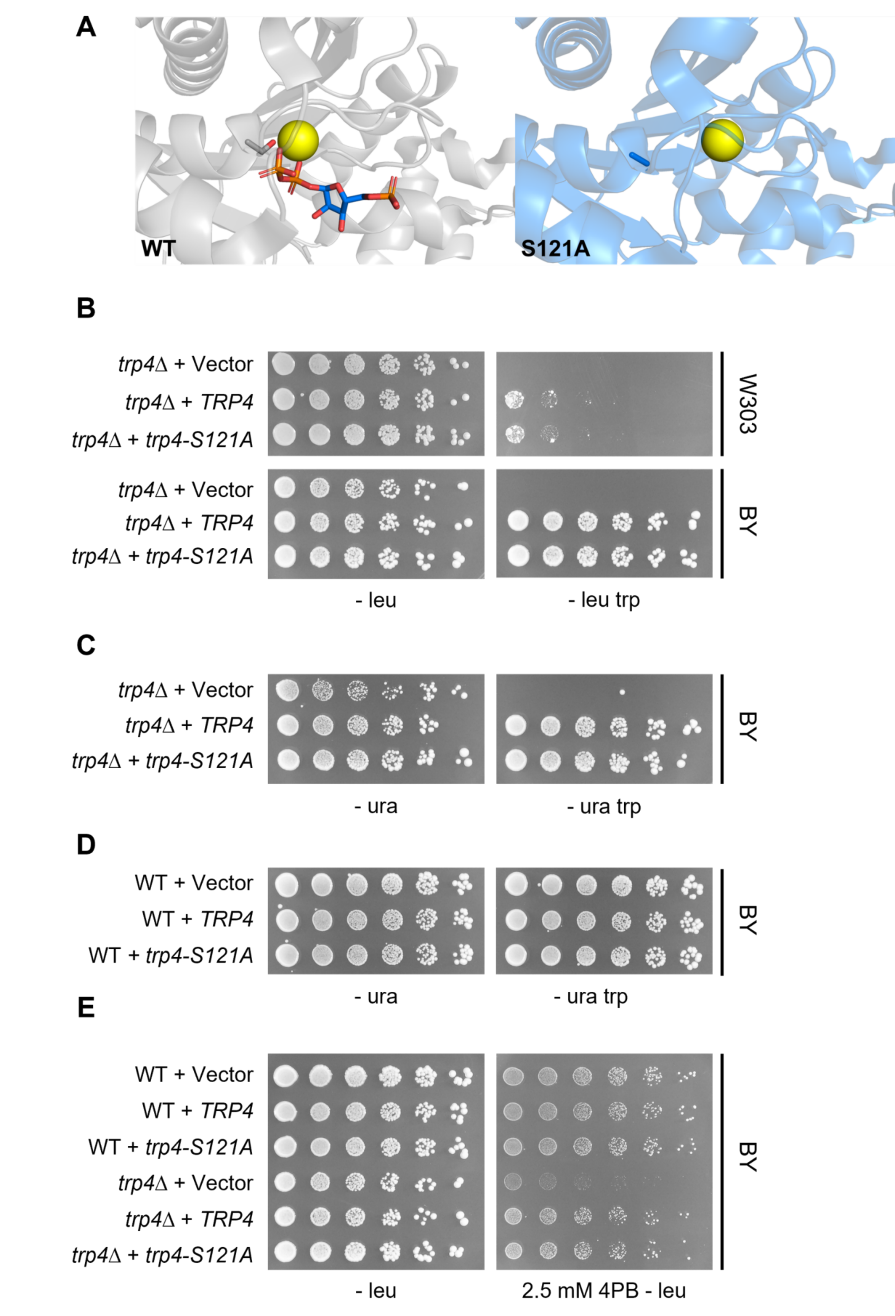
A. Close-up of Trp4 (PDB ID: 7DSJ) and trp4-S121A (PDB ID: 7DSP) active sites. Serine 121 (grey sticks) is required for coordination of Mg ^2+^ (yellow) which coordinates the PRPP substrate (multi-colored sticks). Mutation of serine 121 to alanine (blue sticks) results in altered placement of the Mg ^2+^ ion and an inability to bind PRPP, according to crystallization studies. B. *trp4-S121A* supports growth on tryptophan deficient media in both W303 and BY backgrounds. Imaged after a 5-day incubation. W303 Strains: WT (LPY 23157), *trp4∆* (LPY 23426). BY Strains: WT (LPY 6495), *trp4∆* (LPY 23437). Plasmids: Vector (pLP 400), *TRP4* (pLP 3567), *trp4-S121A* (pLP 3568). C. *trp4-S121A* rescue is not plasmid marker-specific as similar growth patterns are also observed with a *URA3* selectable marker. Assays were imaged after 5 days. Strains: WT (LPY 6495), *trp4∆* (LPY 23437). Plasmids: Vector (pLP 126), *TRP4* (pLP 3571), *trp4-S121A* (pLP 3572). D. The *trp4-S121A* allele does not display dominant phenotypes in WT strains. Images were captured after a 5-day incubation. Strains: WT (LPY 6495), *trp4∆* (LPY 23437). Plasmids: Vector (pLP 126), *TRP4* (pLP 3571), *trp4-S121A* (pLP 3572). E. *trp4-S121A* rescue is comparable to *TRP4* in response to 4PB. Imaged after a 5-day incubation. Strains: WT (LPY 6495), *trp4∆* (LPY 23437). Plasmids: Vector (pLP 400), *TRP4* (pLP 3567), *trp4-S121A* (pLP 3568).

## Description

All cellular life relies upon the synthesis and breakdown of amino acids for the creation of cellular proteins and inputs to metabolic and other regulatory processes. As such, amino acid biosynthetic enzymes are some of the most ancient and fundamental proteins.

In recent years, a class of multi-functional proteins known as moonlighters has expanded understanding of how biological pathways are connected, regulated, and evolve (Jeffery 2014, 2015). Our interest was first drawn with the discovery of chromatin-related functions for amino acid biosynthetic enzymes including homocitrate synthase, glutamate dehydrogenase, and several enzymes with roles in threonine metabolism (Scott and Pillus 2010; Su and Pillus 2016; Chik et al. 2024). To build on this work, an *in silico* screen was used to identify candidates that may function as moonlighting proteins, with a focus on those involved in chromatin regulation (Su and Pillus 2016). Anthranilate phosphoribosyltransferase, encoded by *TRP4* (Furter et al. 1986), met the key criteria of being an amino acid metabolizer with reported nuclear localization (Huh et al. 2003), and was identified within the second tier of the screen.

The structure of *Saccharomyces cerevisiae* Trp4 has been solved in both its apo form and in complex with its substrate phosphoribosyl pyrophosphate (PRPP) (Wu et al. 2021). In addition, two active site mutant proteins, trp4-S121A and trp4-G141N, have been crystallized to determine the functional contributions of these residues. In all cases, the proteins form a dimer mediated through N-terminal interactions. The dimeric state of Trp4 has also been verified *in vitro* via analytical ultracentrifugation studies (Hommel et al. 1989).

Serine 121 is a conserved active site residue, found in 149 out of 150 analyzed sequences across multiple biological kingdoms including Fungi, Bacteria, Archaea, and Plantae, suggesting its functional significance (Berezin et al. 2004). Crystallographic analysis reveals that the Ser121 side chain directly coordinates the Mg ^2+^ ion in the active site, which mediates PRPP binding. In contrast to WT Trp4, the trp4-S121A mutant does not exhibit clear PRPP density in its active site making it a prime candidate to evaluate Trp4 functions independent of tryptophan biosynthetic activity ( Figure 1A ).

Our general strategy in evaluating potential chromatin moonlighting proteins is to construct a null mutant allele in a reporter strain engineered to probe chromatin silencing functions (Roy and Runge 2000) and evaluate transcriptional silencing and other DNA-mediated processes, including DNA repair and recombination. In parallel, structural and experimental data are used to identify alleles or critical residues contributing to catalytic activity. Mutation of these sites allows us to address whether catalytic activity contributes to chromatin-based functions or if the enzymes were ‘moonlighting’ with more than one distinct function.

In assessing *trp4∆* , tryptophan auxotrophy was confirmed in the reporter strain (Roy and Runge 2000) in the W303 background (Thomas and Rothstein 1989), but no reproducibly overt silencing or DNA damage-related phenotypes were found. However, an unexpected result was observed when assessing the function of the proposed catalytically inactive mutant, *trp4-S121A* (Wu et al. 2021). We constructed the allele on a *LEU2* -marked centromeric plasmid and transformed it into W303 strains in the silencing reporter background. Surprisingly, both WT and mutant trp4-S121A proteins supported growth of the null mutant on media lacking tryptophan ( Figure 1B ). To address any strain-background effects, independent transformants were generated in the BY background (Brachmann et al. 1998) and the same results were observed ( Figure 1B ). The reduced growth of W303 strains compared to BY on tryptophan-deficient media is expected, as W303 strains bear the *trp1-1* allele, whereas&nbsp;BY strains are *TRP1.* Further, to evaluate whether there were any effects related to the plasmid selectable marker, we constructed the plasmids independently in a *URA3* -centromeric plasmid. Results with both selectable-marker plasmids were comparable ( Figure 1C ). Because of Trp4’s reported dimeric nature, we also considered the possibility that alone, *trp4-S121A* might support function, but might have dominant interfering effects in a WT strain where *TRP4* was intact. However, no such effects were observed, as full growth was maintained in the *trp4-S121A-* transformed WT strain on medium lacking tryptophan ( Figure 1D ).

Although Trp4 is best known for its role in biosynthesis, we considered the report that *trp4∆* strains have sensitivity to 4-phenylbutyrate (4PB) (Liu et al. 2004). This is a common industrial compound, which for more than four decades has been used in a diverse array of clinical settings (reviewed in (Kolb et al. 2015; Bobat et al. 2025)). The proposed biological mechanisms of 4PB activity are correspondingly diverse, ranging from influences on histone modification and protein chaperone activity, to a demonstrated role in amino acid transport in yeast, with a particularly strong effect on tryptophan transport (Grzanowski et al. 2002; Liu et al. 2004). Indeed, *trp4∆* mutants were exceptionally sensitive to 4PB inhibition in kinetic transport and growth endpoint assays (Liu et al. 2004). We confirmed this sensitivity and observed that growth on 4PB could be restored in *trp4∆* strains transformed with *TRP4* or *trp4-S121A* , but not vector alone ( Figure 1E ).

Thus, our data show that *trp4-S121A* promotes activity required for tryptophan synthesis, that the mutant does not show dominant interference with WT activity, and in independent phenotypic assessment, that it supports resistance to 4PB, a function dependent on anthranilate phosphoribosyltransferase.

We note that these *in vivo* results stand in contrast to expectations raised on the basis of previous structural and biochemical analyses using purified protein. Thus, whereas structural data are invaluable for observing enzyme-substrate interactions, protein crystallization may not capture the full dynamic range of enzymes, which can be revealed by *in vivo* studies. Because crystallization inherently relies on the formation of multiple repeating units, less favorable or weak interactions may not be observed in the final structure under specific crystallization conditions.

Our data show that yeast expressing *trp4-S121A* remain viable on tryptophan-deficient media. We hypothesize that the mutant can support potentially suboptimal PRPP binding in a manner sufficient to support cellular viability. Kinetic analysis of purified trp4-S121A protein and mutational and functional analyses of other residues contributing to Mg ^2+^ coordination would shed additional light on the mechanistic profile of the enzyme.

## Methods

Yeast growth, strains, and plasmid methods

Yeast cells were grown under standard conditions at 30˚C, using either rich YPAD medium, or media to select for the *LEU2* or *URA3* plasmids. The *TRP4* gene was cloned by amplification from genomic DNA and inserted into CEN vectors (Sikorski and Hieter 1989) as an *Apa* I- *Sac* II fragment ( *LEU2* , pRS315) or *Xho* I- *Sac* II fragment ( *URA3* , pRS316). The *trp4-S121A* mutant was constructed by site-directed mutagenesis of the wild-type sequence. Full plasmid sequences were confirmed through nanopore sequencing provided by plasmidsaurus.com.

As a ‘ *caveat emptor’* side note, we initially worked to construct the *trp4-S121A* mutant via CRISPR-mediated mutagenesis in a protocol adapted from (Chik et al. 2024). Transformants were abundant and we thus opted to perform genomic sequencing on only those candidates that were tryptophan auxotrophs. When trp- candidates were sequenced from multiple, independent transformations, they contained the desired mutation, but all had secondary frameshift or missense mutations. We now recognize that our initial assumptions about a critical *in vivo* role for Ser121 were incorrect, as demonstrated by the independent, plasmid-based experiments.

Growth assays

Cells were grown in liquid YPAD or defined drop-out media at 30˚C for 1-2 days and normalized to A _600_ of 1.0. Five-fold serial dilutions were plated onto solid media and images were captured after 3-5 days of growth. 4PB (Cayman Chemical, Ann Arbor MI) was prepared as an aqueous stock, filter-sterilized, and added at the indicated concentration to media that had been cooled after autoclaving.

## Reagents

Yeast strains used in this study

**Table:** 

Strain	Description	Genotype	Reference
LPY 23157	W303 Triple Reporter (WT)	*MATα* *ade2-1 can1-100 his3-11 leu2-3 trp1-1 ura3-1 hmr::TRP1 rDNA::ADE2CAN1 VRTEL::URA3*	Derived from LPY 4654 from (Roy and Runge 2000)
LPY 23426	W303 *trp4∆* with telomeric marker	*MATα* *ade2-1 can1-100 his3-11 leu2-3,112 trp1-1 ura3-1 VRTEL::URA3 trp4::kanMX*	This Study
LPY 6495	BY 4741 (WT)	*MAT* a *his3Δ1* *leu2Δ0 met15Δ0* *ura3Δ0*	(Brachmann et al. 1998)
LPY 23437	BY *trp4∆*	*MAT* a *his3Δ1* *leu2Δ0* *met15Δ0 ura3Δ0* *trp4::kanMX*	(Winzeler et al. 1999)

Plasmids used in this study

**Table:** 

Plasmid	Description	Reference
pLP 400	pRS315-CEN- *LEU2*	(Sikorski and Hieter 1989)
pLP 3567	pRS315-CEN- *LEU2-TRP4*	This study
pLP 3568	pRS315-CEN- *LEU2-trp4-S121A*	This study
pLP 126	pRS316-CEN- *URA3*	(Sikorski and Hieter 1989)
pLP 3571	pRS316-CEN- *URA3* - *TRP4*	This study
pLP 3572	pRS316-CEN- *URA3- trp4-S121A*	This study

Oligonucleotides for cloning and mutagenesis

**Table:** 

Oligo	Purpose	Sequence
oLP 2561	Amplification of *TRP4* genomic DNA for cloning	5’–GCCAGTCAGCGGTTTATTTTATGC–3’
oLP 2562	Amplification of *TRP4* genomic DNA for cloning	5’–CAAGGGCTGAGACATTGGC–3’
oLP 2545	Used to construct *trp4∆::kanMX* strain from LPY 23437 genomic DNA	5’–TTAGACACATGACATAGGC–3'
oLP 2546	Used to construct *trp4∆::kanMX* strain from LPY 23437 genomic DNA	5’–GTGTGGACTGTAACTAACGAAG–3’
oLP 2565	Forward primer for site directed mutagenesis to construct *trp4-S121A* , for pLP3568	5’–GGACAGAATACTTTTAATGTTGCCACGTCTGCTGCTATCG-3’
oLP 2566	Reverse primer for site directed mutagenesis to construct *trp4-S121A* , for pLP3568	5’–CGATAGCAGCAGACGTGGCAACATTAAAAGTATTCTGTCC–3’
